# Impairment of Membrane Repolarization Accompanies Axon Transport Deficits in Glaucoma

**DOI:** 10.3389/fnins.2019.01139

**Published:** 2019-11-01

**Authors:** Rachel A. Fischer, Michael L. Risner, Abigail L. Roux, Lauren K. Wareham, Rebecca M. Sappington

**Affiliations:** ^1^Department of Pharmacology, Vanderbilt University, Nashville, TN, United States; ^2^Vanderbilt Eye Institute, Vanderbilt University Medical Center, Nashville, TN, United States; ^3^Department of Ophthalmology and Visual Sciences, Vanderbilt University School of Medicine, Nashville, TN, United States

**Keywords:** retina, retinal ganglion cells, glaucoma, Na/K-ATPase, potassium, microbead

## Abstract

Glaucoma is a leading cause of blindness worldwide, resulting from degeneration of retinal ganglion cells (RGCs), which form the optic nerve. In glaucoma, axon transport deficits appear to precede structural degeneration of RGC axons. The period of time between the onset of axon transport deficits and the structural degeneration of RGC axons may represent a therapeutic window for the prevention of irreversible vision loss. However, it is unclear how deficits in axon transport relate to the electrophysiological capacity of RGCs to produce and maintain firing frequencies that encode visual stimuli. Here, we examined the electrophysiological signature of individual RGCs in glaucomatous retina with respect to axon transport facility. Utilizing the Microbead Occlusion Model of murine ocular hypertension, we performed electrophysiological recordings of RGCs with and without deficits in anterograde axon transport. We found that RGCs with deficits in axon transport have a reduced ability to maintain spiking frequency that arises from elongation of the repolarization phase of the action potential. This repolarization phenotype arises from reduced cation flux and K+ dyshomeostasis that accompanies pressure-induced decreases in Na/K-ATPase expression and activity. *In vitro* studies with purified RGCs indicate that elevated pressure induces early internalization of Na/K-ATPase that, when reversed, stabilizes cation flux and prevents K+ dyshomeostasis. Furthermore, pharmacological inhibition of the Na/K-ATPase is sufficient to replicate pressure-induced cation influx and repolarization phase phenotypes in healthy RGCs. These studies suggest that deficits in axon transport also likely reflect impaired electrophysiological function of RGCs. Our findings further identify a failure to maintain electrochemical gradients and cation dyshomeostasis as an early phenotype of glaucomatous pathology in RGCs that may have significant bearing on efforts to restore RGC health in diseased retina.

## Introduction

Retinal ganglion cells (RGCs) are the neurons responsible for transmitting visual information from the retina to the brain. Degeneration of RGC axons in glaucoma is the cause of irreversible vision loss for millions of individuals worldwide. Elevated intraocular pressure (IOP) is the primary modifiable risk factor for glaucoma and the current target for therapeutics (Sommer, 1989; Calkins, 2012). RGC degeneration is progressive and irreversible; thus, treatments aimed at lowering IOP only slow the progression of the disease (Sommer, 1989; Calkins, 2012). New therapeutic interventions that address the fundamental mechanisms of RGC degeneration are necessary to halt progression of the disease and preserve vision.

Studies in rodent models of glaucoma indicate that RGC degeneration occurs in a temporal series of events that is common to several neurodegenerative disorders, such as Alzheimer’s disease, Parkinson’s disease, and amyotrophic lateral sclerosis (Ebneth et al., 1998; Braak et al., 2004; Roy et al., 2005; Chevalier-Larsen and Holzbaur, 2006; De Vos et al., 2008; Lee et al., 2011; Calkins, 2012; Millecamps and Julien, 2013). This common path to degeneration, termed axonopathy, is associated with functional deficits in axon transport during early stages of pathology (Calkins, 2012). In animal models of glaucoma, elevated IOP induces significant deficits in axon transport in RGCs (Crish et al., 2010, 2013; Crish and Calkins, 2011; Echevarria et al., 2013; Formichella et al., 2014). As axonopathy progresses, structural degeneration begins in axon terminals and progresses in a retrograde fashion toward the soma (Calkins, 2012). Later stages of degeneration include dendritic pruning and synapse elimination at the soma that is ultimately followed by apoptotic death (Jakobs et al., 2005; Stevens et al., 2007; Crish and Calkins, 2011; El-Danaf and Huberman, 2015; Ou et al., 2016; Risner et al., 2018). Thus, axonopathy in glaucoma results in progressive loss of connection between RGCs and both their pre- and post-synaptic targets (Crish et al., 2010, 2013; Crish and Calkins, 2011; Weitlauf et al., 2014; Risner et al., 2018).

Not surprisingly, several studies indicate that glaucomatous axonopathy is accompanied by changes in the electrophysiological properties of RGCs. Short-term elevations in IOP (1–2 weeks) induce contrasting electrophysiological profiles in RGCs, where response to a preferred light stimulus is stronger, but spontaneous activity is decreased (Risner et al., 2018). Beyond 4 weeks of elevated IOP, both the amplitude and frequency of induced and spontaneous activity is reduced (Chen et al., 2015; Pang et al., 2015; Risner et al., 2018). This is accompanied by increased depolarization of the resting membrane potential and increased variability in membrane noise (Chen et al., 2015; Pang et al., 2015; Risner et al., 2018). These previous studies highlight important changes in RGC physiology happening during glaucoma pathology prior to physical degeneration of RGCs. However, it is unclear how electrophysiological aberrations relate to axon transport deficiencies evident in the early stages of axonopathy (Risner et al., 2018). This stage of disease progression is particularly important, as the period of time between onset of functional deficits and the physical degeneration of RGCs could represent a potential therapeutic window to interrupt axonopathy prior to irreversible, structural loss.

Here, we examined the electrophysiological signature of individual RGCs in glaucomatous retina with respect to their axon transport facility. Utilizing the Microbead Occlusion Model of murine ocular hypertension, we performed electrophysiological recordings of RGCs with and without deficits in anterograde axon transport. Our data indicate that microbead-induced IOP elevation elongates the repolarization phase of the action potential, which is accompanied by reduced capacity to maintain spiking frequency. This repolarization phenotype also coincides with decreased expression of Na/K-ATPase. *In vitro* studies with purified RGCs indicate that elevated pressure reduces cation flux and alters K^+^ homeostasis. These changes in cation homeostasis are accompanied by early internalization of Na/K-ATPase. Pharmacological reversal of this internalization stabilizes cation flux and prevents K^+^ dyshomeostasis. Conversely, pharmacological inhibition of the Na/K-ATPase is sufficient to replicate pressure-induced cation influx and repolarization phase phenotypes in healthy RGCs. These studies suggest that impairment of electrophysiological function in RGCs accompanies deficits in axon transport in this glaucoma model. This electrophysiological impairment appears to arise from a failure to maintain electrochemical gradients and cation dyshomeostasis, which may be an early phenotype of glaucomatous pathology in RGCs.

## Materials and Methods

### Microbead Occlusion Model

Mice were housed in accordance with NIH guidelines and maintained on a 12-h light/dark cycle with free access to food and water. All experiments were approved by the Institutional Animal Care and Use Committee of Vanderbilt University Medical Center. Male C57Bl/6 mice were obtained from Charles River Laboratories (Wilmington, MA, United States). IOP elevation was induced in 1-month-old C57Bl/6 mice, using the microbead occlusion model, as previously described (Crish et al., 2010; Sappington et al., 2010; Echevarria et al., 2016, 2017; Wareham et al., 2018). Briefly, animals were anesthetized with isoflurane and received bilateral injections of 1.5-μl sterile 15 μm polystyrene beads (1 × 10^6^ microbeads/ml; Cat# F8844, Life Technologies, Carlsbad, CA, United States). Control mice received bilateral injections of an equal volume of saline. IOP elevation lasted 4 weeks, at which point the animals were sacrificed. IOP was measured in awake, behaving mice, using a TonoLab tonometer (TonoLab; Reichert, Depew, NY, United States; Echevarria et al., 2013; Ou et al., 2016). IOP was determined as the mean of 10 individual measurements. Prior to initial microbead or saline injections, baseline IOP was recorded for 3 consecutive days. Following injections, IOP was recorded three times a week throughout the 4 week experiment. Mean IOP with standard deviations are provided for each dataset in result text. For each dataset, microbead injection increased mean IOP by approximately 25%, as compared to naïve or saline-injected eyes (*p* < 0.01 for all).

### Electrophysiology

Forty-eight hours prior to electrophysiology experiments, mice received a bilateral, intravitreal injection of fluorophore-conjugated cholera toxin beta subunit (CTB) to label RGCs (Crish et al., 2010; Echevarria et al., 2017). Whole-cell recording was performed, as previously described (Duncan et al., 2018; Risner et al., 2018). Under dim red light (630 nm, 800 μW/cm^2^, Ushio FND/FG), animals were euthanized by cervical dislocation, and retinas were dissected out of the orbit. Whole retinas were mounted onto a physiological chamber and perfused with carbogen-saturated Ames’ medium, supplemented with 20 mM glucose (pH 7.4, 290 Osm), at a rate of 2 ml/min, heated to 32°C (TC-344C, Warner Instruments). Patch pipettes were fabricated from thick-walled borosilicate glass and heat-pulled (P-2000, Sutter Instruments). Pipettes were loaded with (in mM) 125 K-gluconate, 10 KCl, 10 HEPES, 10 EGTA, 4 Mg-ATP, 1 Na-GTP, and 1 Lucifer Yellow dye (pH 7.35, 287 Osm), and had a resistance between 4 and 8 MΩ. Lucifer Yellow is a fluorescent dye used to fill the cells during recordings for later identification with fluorescent imaging. We targeted RGCs with large somas (>15 μm in diameter) for whole-cell recording. Whole-cell voltage signals were amplified (MultiClamp 700B, Molecular Devices) and digitized at a sampling rate of 50 kHz (Digidata 1550A, Molecular Devices). Throughout each experiment, access resistance was continually monitored and was less than 30 MΩ.

We measured spontaneous activity under normal extracellular solution conditions for 1–2 min (3 mM KCl). Then, we increased [K^+^] of the extracellular bath solution to 13 mM by adding additional KCl. Once the cell’s response stabilized, the bath solution was changed to the normal solution for at least 10 min to allow the tissue to recover. Afterward, extracellular [K^+^] was increased to 23 mM by addition of KCl. After the cell’s potential stabilized, we exchanged the high [K^+^] solution back to normal solution and thoroughly washed out the chamber prior to further experiments.

In a separate set of experiments, we performed whole-cell current-clamp (0 pA) recordings from RGCs while applying ouabain (10 or 20 μM, Cat# O3125, Sigma Aldrich, St Louis, MO) via a wide-bore pipette attached to pneumatic microinjection device (PicoSpritzer II, General Valve Corp.). The “puff” pipette was positioned approximately 150–200 μm from the RGC soma and 10 or 20 μM ouabain, which was dissolved in extracellular medium, and was administered for 30 ms at 10–12 psi while recording the drug-induced spike activity and membrane potential. After each experiment, the drug was thoroughly washed out with extracellular solution.

Following the completion of all electrophysiology experiments, retinas were fixed overnight in 4% paraformaldehyde. Fluorescent confocal microscopy was used to visualize CTB-594 labeling of RGCs and any cells labeled with Lucifer Yellow dye, using an Olympus FV-1000 inverted confocal microscope (Olympus, Tokyo, Japan).

### RNA Sequencing

Whole, intact retina was dissected from microbead occlusion model mice following sacrifice. Immediately following dissection, RNA was extracted from retina using TRIzol (Invitrogen, Cat# 15596026) and treated with deoxyribonuclease (DNase) I (Worthington, Cat# LS006333). Experiments were performed through the Vanderbilt Technologies for Advanced Genomics core at Vanderbilt University Medical Center. DNase-treated total RNA quality was assessed using the 2100 Bioanalyzer (Agilent Technologies). Samples with integrity values greater than 6 were used to generate polyA (mRNA)-enriched libraries, using stranded mRNA sample kits with indexed adaptors (New England BioLabs, Ipswich, MA, United States). Library quality was assessed using the 2100 Bioanalyzer (Agilent Technologies) and libraries were quantitated using KAPA Library Quantification Kits (KAPA Biosystems, Wilmington, MA, United States). Pooled libraries were subjected to 100-bp paired-end sequencing according to the manufacturer’s protocol (Illumina NovaSeq6000). Bcl2fastq2 Conversion Software (Illumina, San Diego, CA, United States) was used to generate de-multiplexed Fastq files. Analysis of RNAseq results was performed through the Vanderbilt Technologies for Advanced Genomics Analysis and Research Design core at Vanderbilt University. Reads were aligned to the GENCODE GRCm38.p5 genome using STAR v2.5.3a. GENCODE vM12 gene annotations were provided to STAR to improve the accuracy of mapping. Quality control on raw reads was performed using FastQC. FeatureCounts v1.15.2 was used to count the number of mapped reads to each gene. Significantly differential expressed genes with adjusted *p* value < 0.05 and absolute fold change>2 were detected by DESeq2 v1.14. Data are reported as fold change (log scale) and percent change in RNA transcript levels between saline and microbead retina. Data have been deposited in NCBI’s Gene Expression Omnibus and are accessible through GEO series accession number GSE116915^1^.

### Immunohistochemistry

We assessed expression and localization patterns of total and α1 subunit Na/K-ATPase expression using immunohistochemistry (Sappington and Calkins, 2006, 2008; Sappington et al., 2006, 2009; Lee et al., 2015). Immunohistochemistry experiments were done on longitudinal paraffin-embedded retina sections of whole eyes from microbead-injected, saline-injected, and naïve mice. A separate set of experiments was also done in RGC cultures exposed to ambient or elevated pressure for 4 or 48 h. Cells were fixed in 4% paraformaldehyde (Cat# 15714-S, Electron Microscopy Sciences, Hatfield, PA, United States) for 15 min at room temperature and washed with 1 × PBS. Samples (fixed, cultured cells and retinal sections) were incubated in blocking solution containing 5% normal horse serum (NHS; Life Technologies) and 0.1% Triton-X (Fisher Scientific) in 1 × PBS. Samples were then incubated overnight at 4°C in primary antibody solution (3% NHS and 0.1% Triton X-100 diluted in 1 × PBS) containing rabbit anti-Total Na/K-ATPase (2 μg/ml, Cat# ab58475, Abcam, Cambridge, United Kingdom), rabbit anti-α1 Na/K-ATPase (2.85 μg/ml, Cat# ANP-001, Alomone, Jerusalem, Israel); and mouse anti-β-Tubulin III (2 μg/ml, Cat# 801201, BioLegend, London, United Kingdom). Following 1 × PBS washes, samples were incubated for 2 h at room temperature in a secondary antibody solution containing 1% NHS, 0.1% Triton X-100, and either 647-donkey anti-mouse (7.5 μg/ml; Cat# 715-606-150, Jackson ImmunoResearch) or 488-donkey anti-rabbit (7.5 μg/ml; Cat# 711-546-152, Jackson ImmunoResearch) in 1 × PBS. Samples were counterstained with the nuclear stain DAPI (50 μg/ml; Cat# D1306, Life Technologies) and coverslipped in aqueous mounting media (Southern Biotech, Birmingham, AL, United States). Immunolabeling was imaged at 40 × using an Olympus FV-1000 inverted confocal microscope (Olympus, Tokyo, Japan). Images compared between control and experimental groups were collected at the same time, under the same imaging parameters. Mean fluorescence intensity was calculated using the FIJI Color Histogram tool (ImageJ).

### Purified Primary Cultures

Primary cultures of purified RGCs were prepared as previously described (Sappington et al., 2006, 2009, 2015; Lee et al., 2015). Briefly, eyes (*n* = 16/preparation) from postnatal day 2–4 Sprague-Dawley rats were enucleated. The retina of each eye was dissected and retinal tissue was dissociated. RGCs were purified by immunomagnetic separation, using mouse anti-rat Thy-1.1/Cd90 IgG antibody (5 μg/ml, Cat# 554895, BD Biosciences, San Jose, CA, United States) and metallic microbeads conjugated to anti-mouse IgG secondary antibody (Cat# 130-047-102, Miltenyi Biotec, Auburn, CA, United States). RGCs were plated in 8-well chamber slides coated overnight with laminin (0.01 mg/ml; Cat# L6274, Sigma). RGCs were grown in serum-free, Neurobasal A media (Cat# 21103049, Gibco, Carlsbad, CA, United States), containing the following supplements: 2% B27 (Cat# 17504044, Gibco), 1% N2 (Cat# 17502048, Gibco), 2 mM L-glutamine (Cat# G7513, Sigma), 100 μM inosine (Cat# 58-63-9, Sigma), 0.1% gentamicin (Cat# 15710-064, Gibco), 50 ng/ml brain-derived nerve growth factor (Cat# PHC7074, Gibco), 20 ng/ml ciliary neurotrophic factor (Cat# PRC7015, Gibco), and 10 ng/ml basic fibroblast growth factor (Cat# 13256-029, Gibco). Experiments were performed on RGCs approximately 1 week after plating. Final *n*’s for culture experiments were sampled from 3 to 4 individual platings on 2–3 different cell isolation days. The following drugs used for cell culture experiments were dissolved in the culture media and used at designated concentrations: ouabain (20 μM, Cat# O3125, Sigma Aldrich), bisindolylmaleimide I (10 μM, Cat# 176504-36-2, Millipore Sigma, Burlington, MA, United States), and MG-132 (20 μM, Cat# 133407-82-6, Millipore Sigma).

### Elevated Hydrostatic Pressure

Primary cultures of purified RGCs were maintained at ambient or at + 70 mmHg hydrostatic pressure, for 4 or 48 h, as previously described (Sappington and Calkins, 2006, 2008; Sappington et al., 2006, 2009; Lee et al., 2015). Briefly, a humidified pressure chamber equipped with a regulator and a gage was placed in a 37°C incubator; a mixture of 95% air and 5% CO_2_ was pumped into the chamber to obtain a pressure of +70 mmHg (9% increase above atmospheric pressure) that was maintained by the regulator. For ambient pressure experiments, cells were kept in a standard incubator.

### TUNEL Reactivity

In RGC cultures exposed to ambient or elevated pressure for 4 or 48 h, we measured apoptosis with TdT-mediated dUTP-X nick end labeling (TUNEL; Cat# 12156792910, Roche, Basel, Switzerland). Labeling was performed according to the manufacturer’s specifications and as previously described (Gavrieli et al., 1992; Gorczyca et al., 1993; Sappington et al., 2006; Lee et al., 2015). Cells were counterstained with mouse anti-β-Tubulin III (2 μg/ml, Cat# 801201, BioLegend) to label RGCs for confirmation of cell type and DAPI for quantification of total cell density. To quantify % TUNEL+ cells, 20 × images were taken using a Roper Scientific black and white camera (Photometrics, Tucson, AZ, United States) mounted to a Nikon Ti microscope (Nikon Instruments, Melville, NY, United States). Images compared between control and experimental groups were collected at the same time, under the same imaging parameters. Five images were taken per well, across four wells per condition. Total numbers of β-Tubulin III+/DAPI+ and TUNEL+/β-Tubulin III+/DAPI+ were counted for each image and summed for each well. Data are shown as % TUNEL+ cells relative to the total number of β-Tubulin III+/DAPI+ cells, normalized as the percent change from control (ambient pressure).

### Lactate Dehydrogenase Assay

In RGC cultures maintained at ambient or elevated pressure for 4 or 48 h, we measured cell toxicity, which is related to necrotic cell death, using a lactate dehydrogenase (LDH) assay (Cat# G1780, Promega, Madison, WI, United States). The assay was performed according to the manufacturer’s specifications and as previously described (Sappington et al., 2006). Briefly, culture supernatant was collected following pressure elevation and immediately frozen at −80°C. The concentration of LDH in culture supernatants was determined by enzymatic reaction and measurement of optical density (OD) at 490 nm. All samples were run in triplicate and OD levels were averaged. Background OD levels were obtained from blank media samples and subtracted from OD levels of experimental samples. Data are shown as OD at 490 nm with background subtracted, normalized as the percent change from control (ambient pressure).

### Thallium Flux Imaging

We assessed ion channel activity in live RGC cultures exposed to 4 or 48 h of ambient or elevated pressure by thallium flux imaging, as previously described (Fischer et al., 2018). Thallium flux indirectly measures inward ion flux, through the fluorescence intensity of Thallos dye. Briefly, cells were loaded with Thallos-AM dye (0.5 μg/μl, Cat# 0902, TEFlabs, Austin, TX, United States), generously provided by Dave Weaver of Vanderbilt University, by incubating with the dye for 30 min at 37°C. Following dye loading, cells were washed with fresh media. Using live cell fluorescence microscopy, the baseline fluorescence of the Thallos dye was recorded for continuously 6–8 s (images taken every 1 s). One millimolar thallium (TI^+^) solution was then added to cell culture media and live imaging was performed continuously for 45 s (images taken every 1 s). Fluorescence intensity was calculated for each image using Nikon NIS-Elements software. Data were plotted as normalized fluorescence intensity at each time point.

### Inductively Coupled Plasma Mass Spectrometry

In supernatants from RGC cultures exposed to 4 or 48 h ambient or elevated pressure, we measured the extracellular concentration of K^+^, using inductively coupled plasma mass spectrometry (ICP-MS). Experiments were performed through the Mass Spectrometry Research Center at Vanderbilt University. Culture supernatants were diluted 1000 × with Milli-Q water (Millipore, Milli-Q synthesis advantage A-10, Millipore Corp., Burlington, MA, United States) and prepared alongside a calibration curve made utilizing the same water. Na^+^ and K^+^ standards (Fluka, Sigma-Aldrich, L’Isle-d’Abeau Chesnes, Saint-Quentin-Fallavier, France) were serially diluted for the calibration curve, which ranged from 10 ppm to 1 ppb. Samples and standards were immediately analyzed by ICP-MS on an Agilent model 7700_*x*_ (Agilent Technologies, Santa Clara, CA, United States). Each sample was introduced to the instrument manually, without use of an autosampler, and the probe was rinsed in 2% nitric acid (Optima grade, Fisher Scientific Co., Pittsburgh, PA, United States) between each sample. Three blank samples of pure Milli-Q water between introduction of standards and samples confirmed the absence of background K^+^. The data were analyzed by the offline data analysis package (Agilent Technologies), where the calibration curve was plotted linearly and both *R*^2^ values, as well as detection limits, were calculated. The calculated concentrations of each sample were multiplied by 1000 to account for the initial dilution factor. Data are shown as average K^+^ concentration in parts per million.

### Statistical Analysis

All statistical tests were conducted with SigmaPlot (Systat Software Inc., San Jose, CA, United States). Experimental groups were compared within time points by Student’s *t* test. Normality (Shapiro-Wilk) and equal variance were also assessed for each comparison. Comparisons between time points within groups were assessed by one-way ANOVA followed by pairwise comparison by either the Tukey or Dunn’s method. Significant comparisons were marked by brackets and asterisks. For all analyses, *p* ≤ 0.05 was considered statistically significant.

## Results

### Impairment of Electrochemical Gradients in Glaucomatous RGCs

**FIGURE 1:**
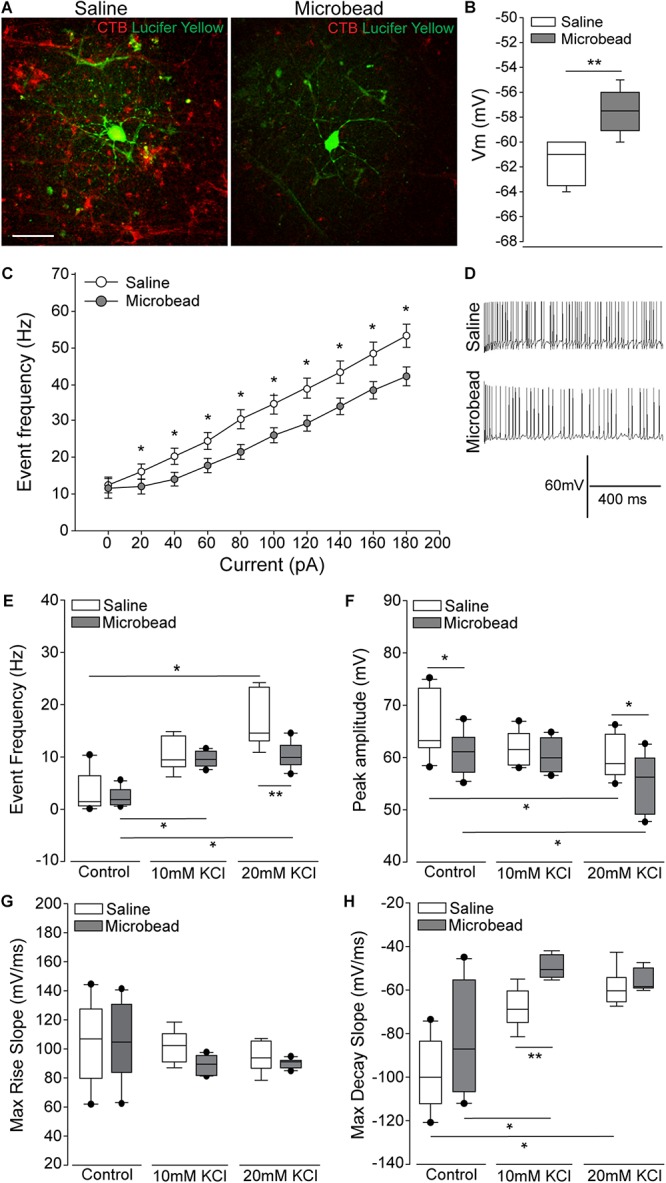
Retinal ganglion cells (RGCs) with deficient axon transport exhibit decreases in induced spiking frequency that result from delayed repolarization. **(A)** Representative confocal micrographs of RGCs in whole retina following electrophysiological recordings from saline- (left) and microbead-injected (right) eyes. RGCs are CTB-traced (red) and filled with lucifer yellow (green). Scale bar = 50 μM. **(B)** Mean resting membrane potential (Vm) of RGCs from saline- (white) and microbead-injected (gray) retina. ^∗∗^*p* < 0.001 and error bars represent SEM. *n*(saline) = 27 cells (from 14 retina), *n*(microbead) = 34 cells (from 11 retina). **(C)** Frequency of spiking of RGCs from saline- (white) and microbead-injected (gray) retina following current stimulation in 20 pA steps for 1 s from 0 to 180 pA. Mean event frequency (Hz) is displayed at each current step. ^∗^*p* < 0.05. *n*(saline) = 26 cells (from 14 retina), *n*(microbead) = 26 cells (from 11 retina). **(D)** Representative traces of spiking with 180 pA stimulation. **(E)** Frequency of spontaneous spiking of RGCs from saline- (white) and microbead-injected (gray) retina in control bath, or bath containing 10 or 20 mM KCl. Data are displayed as mean event frequency (Hz) across cells for each condition. ^∗^*p* < 0.05, ^∗∗^*p* < 0.005. *n*(saline) = 12, *n*(microbead) = 10, *n*(saline 10) = 9, *n*(microbead 10) = 12, *n*(saline 20) = 9, *n*(microbead 20) = 11 (from *n* = 5 retina/condition). **(F)** Peak amplitude of spontaneous spiking of RGCs. Data are displayed as mean peak amplitude (mV) across cells for each condition. ^∗^*p* < 0.05. *n*(saline) = 11, *n*(microbead) = 11, *n*(saline 10) = 10, *n*(microbead 10) = 12, *n*(saline 20) = 11, *n*(microbead 20) = 17 (from *n* = 5 retina/condition). **(G)** Max rise slope of spontaneous spiking of RGCs. Data are displayed as mean max rise slope (mV/ms) across cells for each condition. *n*(saline) = 14, *n*(microbead) = 13, *n*(saline 10) = 9, *n*(microbead 10) = 10, *n*(saline 20) = 9, *n*(microbead 20) = 10 (from *n* = 5 retina/condition). **(H)** Max decay slope of spontaneous spiking of RGCs. Data are displayed as mean max decay slope (mV/ms) across cells for each condition. ^∗^*p* < 0.05, ^∗∗^*p* < 0.001. *n*(saline) = 10, *n*(microbead) = 12, *n*(saline 10) = 9, *n*(microbead 10) = 9, *n*(saline 20) = 9, *n*(microbead 20) = 9 (from *n* = 5 retina/condition).

To determine the electrophysiological state of RGCs with respect to axon transport facility, we performed whole-cell patch-clamp recordings and cell filling in RGCs with intact and deficient anterograde transport of the neural tracer CTB)(Crish et al., 2010; Formichella et al., 2014). Retina from microbead-injected (IOP = 21 mmHg ± 0.93 mmHg) mice exhibited clusters of RGCs with deficient CTB transport (right panel; Figure 1A), as compared to intact and uniform tracing observed in saline-injected mice (IOP = 14 mmHg ± 0.93 mmHg; left panel; Figure 1A). All recordings in retina from microbead-injected mice were from RGCs with deficient CTB transport. In microbead-injected mice, RGCs possessed a significantly more depolarized resting membrane potential (Vm), compared to RGCs from saline retina (Figure 1B, *p* < 0.001). Current-clamp mode (20 pA/1 s steps; 0–180 pA) revealed significantly lower frequency of action potential firing at each current step, excluding 0 pA, in RGCs from microbead retina, as compared to RGCs from saline retina (Figure 1C, *p* < 0.05). Representative traces of spiking at 180 pA are shown in Figure 1D. Deficiencies in induced spiking frequencies can indicate impairment of hyperpolarization during the post-potential period. This hyperpolarization phase re-establishes electrochemical gradients of ions required for the neuron to fire another action potential.

To determine whether altered ion concentration gradients underlie the reduced spiking frequency and depolarized resting membrane potential of glaucomatous RGCs, we disrupted the extracellular concentration of K^+^ ([K^+^]_*E*_) in the electrophysiological bath. We recorded spontaneous activity of the RGCs in normal, control bath, and bath containing 10 or 20 mM of KCl. In naïve retina, increasing [K^+^]_*E*_ depolarizes the resting membrane potential and induces firing of action potentials in RGCs (Dreyfus et al., 1986). In saline retina (white bars), 10 or 20 mM KCl induced a reciprocal increase in the spike frequency of RGCs. This resulted in a significant increase in the event frequency between RGCs in control bath vs. 20 mM KCl (Figure 1E, *p* < 0.05). In microbead retina (gray bars), 10 or 20 mM KCl also significantly increased the spiking frequency (Figure 1E, *p* < 0.05). However, the magnitude of this increase was smaller, resulting in a significantly lower spike frequency in RGCs from microbead retina than in saline retina (Figure 1E, *p* < 0.005). In RGCs from both saline (white bar) and microbead (gray bar) retina, increasing [K^+^]_*E*_ induced a reciprocal decrease in the peak amplitude of spikes that was significantly different between control and 20 mM KCl bath conditions (Figure 1F, *p* < 0.05 for both). However, the amplitude of spikes in RGCs from microbead retina was lower than those in saline retina at control and 20 mM KCl bath conditions (Figure 1F, *p* < 0.05 for both).

To independently evaluate the depolarization and repolarization phases of the action potential, we compared the max rise and decay slope in saline and microbead RGCs following increases in [K^+^]_*E*_. For the depolarization phase, there was no significant difference in the max rise slope between RGCs from saline or microbead retina, at any [K^+^]_*E*_ (Figure 1G, *p* > 0.05). For the repolarization phase, increases in the decay slope toward a value of 0 indicated flattening and slowing of this phase. In RGCs from saline retina (white bars), increased [K^+^]_*E*_ induced a reciprocal increase in the max decay slope of the action potential that was statistically significant at 20 mM KCl, as compared to control bath (Figure 1H, *p* < 0.05). In RGCs from microbead retina (gray bars), the decay slope also significantly increased as [K^+^]_*E*_ increased (Figure 1H, *p* < 0.05). KCl-induced increases in the decay slope were greater in RGCs from microbead retina than those from saline retina, which resulted in a significant difference at 10 mM KCl (Figure 1H, *p* < 0.001). These data indicate that RGCs with IOP-induced deficits in axon transport exhibit a compromised ability to maintain induced spiking that is likely due to alterations in the electrochemical gradient impacting the repolarization phase of the action potential.

### Elevated IOP Alters Expression of the Na/K-ATPase

**FIGURE 2:**
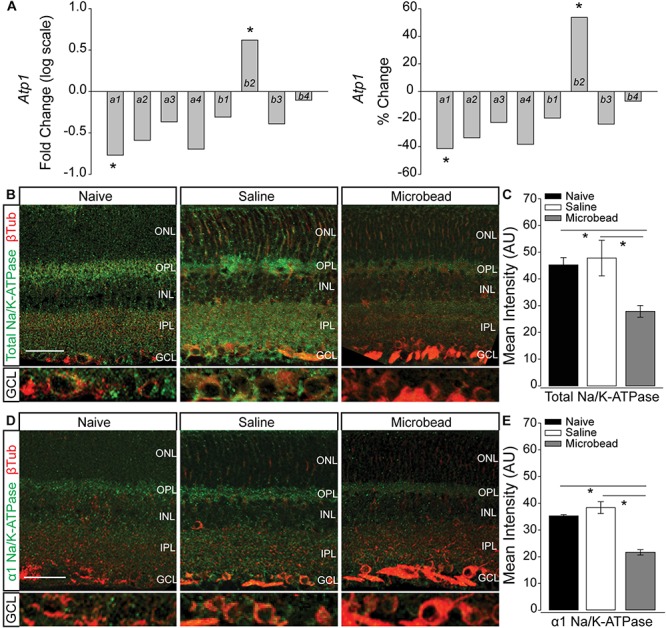
Elevated IOP decreases expression of total and α1 Na/K-ATPase in retina. **(A)** Box plots of fold change (log scale; left) and percent change (right) in expression of the *Atp1* gene family between retina from saline- and microbead-injected mice, as determined by RNA sequencing. *n*(saline) = 5 retinas, *n*(microbead) = 5; ^∗^*p* < 0.005. **(B)** Representative confocal micrographs of total Na/K-ATPase (green) and β-Tubulin III (β-Tub; red) immunolabeling in retinal sections from naïve, saline-, and microbead-injected mice. Bottom panels of each image are a zoom of the ganglion cell layer (GCL). Scale bar = 40 μM. **(C)** Mean intensity of total Na/K-ATPase immunolabeling in retina from naïve, saline-injected, and microbead-injected mice. Asterisk indicates *p* < 0.05 and error bars represent SEM. *n*(naïve) = 8 retinal sections, *n*(saline) = 9, *n*(microbead) = 9 (from *n* = 4 eyes/condition). **(D)** Representative confocal micrographs of α1 Na/K-ATPase (green) and β-Tubulin III (β-Tub; red) immunolabeling in retinal sections from naïve, saline-, and microbead-injected mice. Bottom panels of each image are a zoom of the GCL. Scale bar = 40 μM. **(E)** Mean intensity of α1 Na/K-ATPase immunolabeling in retina sections from naïve, saline-injected, and microbead-injected mice. ^∗^*p* < 0.05 and error bars represent SEM. *n*(naïve) = 4 retinal sections, *n*(saline) = 8, *n*(microbead) = 5 (from *n* = 4 eyes/condition). ONL, outer nuclear layer, OPL, outer plexiform layer, INL, inner nuclear layer, IPL, inner plexiform layer, GCL, ganglion cell layer.

In neurons, including RGCs, the Na/K-ATPase is the primary mechanism for resetting the electrochemical gradient of ions. To determine whether elevated IOP alters expression of the Na/K-ATPase, we examined transcription of the *Atp1* gene family, which is responsible for formation of the Na/K-ATPase. Transcriptional regulation of the *Atp1* gene family was quantified by RNA sequencing in whole retina of C57Bl/6 mice 4 weeks after either saline (IOP = 15 mmHg ± 0.43 mmHg) or microbead (IOP = 21 mmHg ± 0.53 mmHg) injection. Transcriptome analysis revealed a significant IOP-dependent decrease in the transcription of *Atp1a1* by 41.3% and an increase in *Atp1b2* by 53.7% (Figure 2A, *p* < 0.005). There was no significant difference observed in transcription of *Atp1a2, a3, a4, b1, b3*, or *b4* in microbead- versus saline-injected eyes (*p* > 0.05; Figure 2A). *Atp1a1* encodes the α1 isoform of the catalytic subunit, and *Atp1b2* encodes the β2 isoform of the glycoprotein subunit of the Na/K-ATPase. The α1 subunit of the Na/K-ATPase is expressed in RGCs and is responsible for ion exchange (McGrail and Sweadner, 1986, 1989; Wetzel et al., 1999).

Transcriptome analysis in whole retina represents the entire complement of retinal neurons, glia, and vascular elements. To determine the localization of IOP-dependent changes in Na/K-ATPase expression, we performed immunohistochemical staining for total Na/K-ATPase in whole eye sections from naïve, saline-, and microbead-injected mice. Qualitatively, immunolabeling for total Na/K-ATPase (green) was reduced in retina from microbead-injected mice, as compared to both naïve and saline-injected controls (Figure 2B). This decrease in total Na/K-ATPase immunolabeling was particularly dramatic in the ganglion cell layer (GCL; Figure 2B, bottom panel). Quantification of immunolabeling intensity confirmed that elevated IOP in microbead-injected eyes (IOP = 21 mmHg ± 1.1 mmHg) decreased intensity of total Na/K-ATPase by 38 and 42%, as compared to both naïve (IOP = 14 mmHg ± 1 mmHg) and saline-injected controls, respectively (Figure 2C, *p* < 0.05). Since transcriptome analysis revealed decreased expression of the α1 subunit of the Na/K-ATPase, we then evaluated the localization pattern of the α1 subunit, specifically in retina from naïve, saline-injected and microbead-injected mice. Qualitatively, immunolabeling for α1 Na/K-ATPase (green) was reduced in retina from microbead-injected mice, as compared to both naïve and saline-injected controls (Figure 2D), particularly in the GCL (Figure 2D, bottom panel). Quantification of immunolabeling intensity confirmed that elevated IOP decreased intensity of α1 Na/K-ATPase by 39 and 44%, as compared to both naïve and saline-injected controls, respectively (Figure 2E, *p* < 0.05). These combined data suggest that elevated IOP decreases expression of the α1 subunit of the Na/K-ATPase, particularly in the GCL, that leads to an overall reduction of total Na/K-ATPase.

### Short- and Long-Term Pressure Elevation Alters Expression of the Na/K-ATPase in RGCs *in vitr*o

**FIGURE 3:**
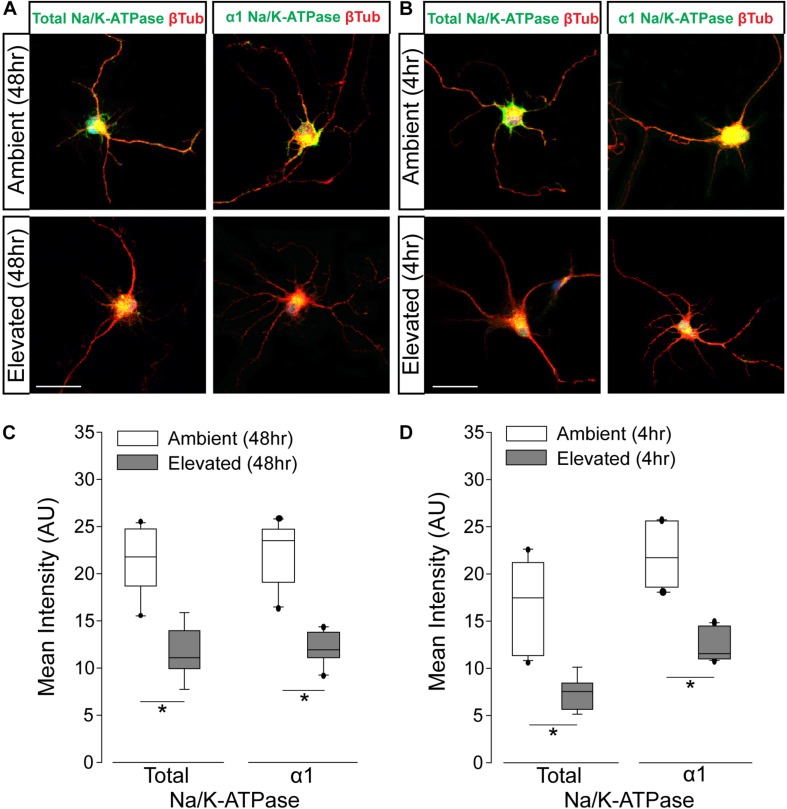
Elevated pressure decreases expression of total and α1 Na/K-ATPase in RGCs *in vitro*. **(A)** Representative fluorescent micrographs of total and α1 Na/K-ATPase (green) and β-Tubulin (red) immunolabeling in primary cultures of purified RGCs exposed to ambient or elevated pressure for 48 h. Scale bar = 40 μm. **(B)** Representative fluorescent micrographs of total and α1 Na/K-ATPase (green) and β-Tubulin (red) immunolabeling in primary cultures of purified RGCs exposed to ambient or elevated pressure for 4 h. Scale bar = 40 μm. **(C)** Mean intensity (arbitrary units; AU) of total (left) and α1 (right) Na/K-ATPase staining in RGC cultures following a 48-h exposure to ambient or elevated hydrostatic pressure. ^∗^*p* < 0.05. Total: *n*(AMB48) = 10 cells, *n*(ELEV48) = 9; α1: *n*(AMB48) = 13, *n*(ELEV48) = 11. **(D)** Mean intensity (arbitrary units; AU) of total (left) and α1 (right) Na/K-ATPase staining in RGC cultures following a 4 h exposure to ambient or elevated hydrostatic pressure. ^∗^*p* < 0.05. Total: *n*(AMB4) = 14 cells, *n*(ELEV4) = 9; α1: *n*(AMB4) = 10, and *n*(ELEV4) = 10.

To verify pressure-induced changes in Na/K-ATPase expression specifically in RGCs, we performed immunocytochemistry staining for total and α1 Na/K-ATPase in primary cultures of purified RGCs from early postnatal rats exposed to either ambient or elevated hydrostatic pressure for 4 or 48 h. As previously reported (Sappington et al., 2006), exposure to +70 mmHg elevated pressure for 48 h induces apoptotic, but not necrotic, death of RGCs, as measured by TUNEL and LDH assays (Supplementary Figure S1). Exposure to only 4 h did not induce either apoptotic or necrotic death of RGCs (Supplementary Figure S1). At ambient pressure, immunolabeling for both total and α1 Na/K-ATPase was most prevalent in RGC soma (top panels; Figures 3A,B). Qualitatively, exposure to both 48 and 4 h of elevated hydrostatic pressure reduces the intensity of immunolabeling for total and α1 Na/K-ATPase in both the soma and neurites (bottom panels; Figures 3A,B). Quantification of immunolabeling intensity per cell revealed that 48 h of elevated hydrostatic pressure decreased total Na/K-ATPase immunolabeling intensity by 45% and α1 Na/K-ATPase by 45%, as compared to 48 h of ambient pressure (Figure 3C, *p* < 0.05). Similarly, 4 h of elevated hydrostatic pressure decreased total Na/K-ATPase by 46% and α1 Na/K-ATPase by 35%, as compared to 4 h of ambient pressure (Figure 3D, *p* < 0.05). These data confirmed that elevated pressure decreases Na/K-ATPase expression in RGCs. Furthermore, significant decreases in Na/K-ATPase expression were noted at 4 h, suggesting that this decrease occurs prior to the initiation of apoptosis and likely occurs, at least initially, via modulation of existing Na/K-ATPase protein.

### Inhibition of Endocytosis and Degradation Pathways Preserves Na/K-ATPase Expression in RGCs Following Short-Term Pressure Exposure

**FIGURE 4:**
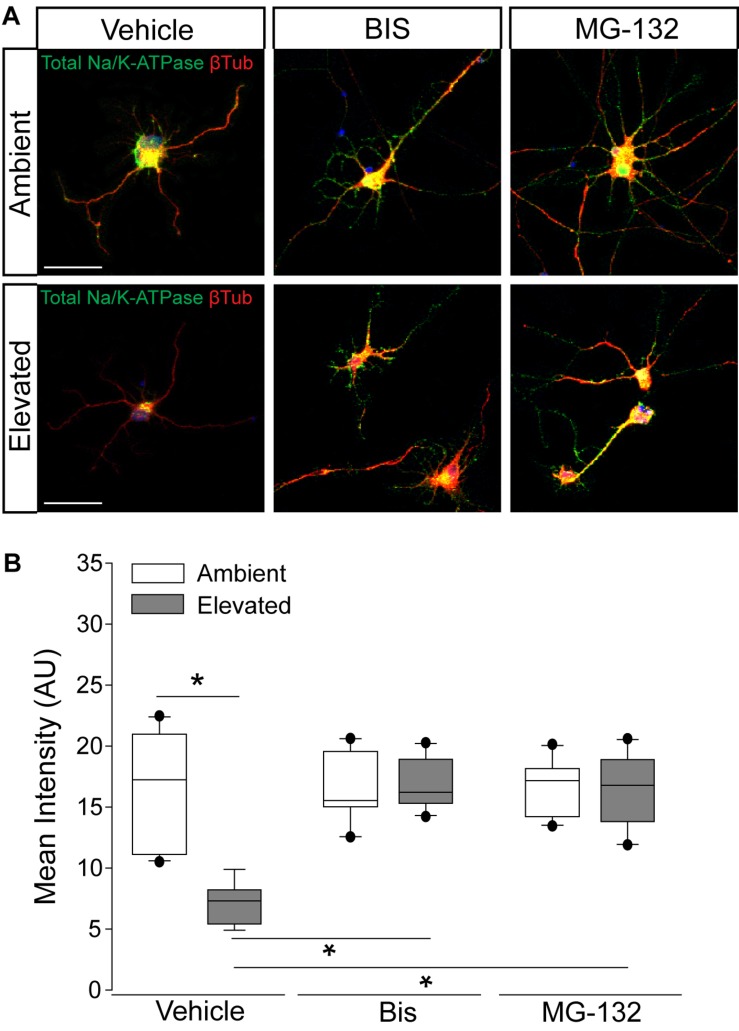
Inhibition of endocytosis and degradation pathways preserves Na/K-ATPase expression in RGCs following short-term pressure exposure. **(A)** Representative confocal micrographs of total Na/K-ATPase (green) and β-Tubulin III (red) immunolabeling in primary, purified RGCs treated with vehicle, 10 μM bisindolylmaleimide, or 20 μM MG-132 and exposed to ambient or elevated pressure for 4 h. Scale bar = 40 μM. **(B)** Mean intensity (arbitrary units; AU) of total Na/K-ATPase staining per cell in each drug and pressure condition. ^∗^*p* < 0.05. *n*(AMB) = 14 cells, *n*(ELEV) = 9; *n*(AMB + BIS) = 11, *n*(ELEV + BIS) = 10; *n*(AMB + MG) = 13, and *n*(ELEV + MG) = 14.

To validate the idea that decreases in Na/K-ATPase expression with short-term pressure exposure is mediated at the protein level, we targeted endocytosis and degradation pathways of the Na/K-ATPase with the PKC inhibitor bisindolylmaleimide and the proteasome inhibitor MG-132. Na/K-ATPase endocytosis is initiated by PKC-mediated phosphorylation of the α1 subunit of the Na/K-ATPase (Lecuona et al., 2009; Magnani et al., 2017). Following endocytosis, the Na/K-ATPase is degraded by the proteasome (Lecuona et al., 2009; Magnani et al., 2017). In other cell types, bisindolylmaleimide-mediated inhibition of PKC prevents endocytosis of the Na/K-ATPase to increase its representation at the plasma membrane (Lecuona et al., 2009). Similarly, inhibition of proteasome activity by MG-132 increases Na/K-ATPase representation on the plasma membrane by inhibiting its degradation (Lecuona et al., 2009). Thus, we treated primary RGC cultures with 10 μM bisindolylmaleimide, 20 μM MG-132, or vehicle during exposure to ambient or elevated hydrostatic pressure for 4 h. We then examined expression and localization of the Na/K-ATPase, using immunocytochemistry. Qualitatively, exposure to 4 h of elevated hydrostatic pressure reduced the intensity of immunolabeling for total Na/K-ATPase in both the soma and neurites (Figure 4A). Consistent with Figure 3, 4 h of elevated pressure decreased immunolabeling of total Na/K-ATPase by 46%, compared to ambient pressure (Figure 4B, *p* < 0.05). In RGC cultures exposed to elevated pressure, treatment with the PKC inhibitor bisindolylmaleimide significantly increased the immunolabeling intensity of total Na/K-ATPase by 46%, as compared to vehicle treatment (Figure 4B, *p* < 0.05). Inhibition of the proteasome with MG-132 significantly also increased the immunolabeling intensity of total Na/K-ATPase by 46% at elevated pressure, as compared to vehicle treatment (Figure 4B, *p* < 0.05). Neither bisindolylmaleimide nor MG-132 altered total Na/K-ATPase immunolabeling in RGC cultures maintained at ambient pressure, as compared to vehicle treatment (Figure 4B, *p* > 0.05). Similarly, there was no significant difference in immunolabeling intensity between bisindolylmaleimide- and MG-132-treated RGCs at elevated pressure (Figure 4B, *p* > 0.05). These data suggest that pressure-induced reductions in Na/K-ATPase expression are initially caused by endocytosis and degradation of existing protein. Furthermore, inhibition of either endocytosis or degradation can prevent, with equal efficacy, this initial pressure-induced reduction in Na/K-ATPase protein representation.

### Elevated Pressure Alters Cation Flux and K^+^ Homeostasis in RGCs *in vitr*o

**FIGURE 5:**
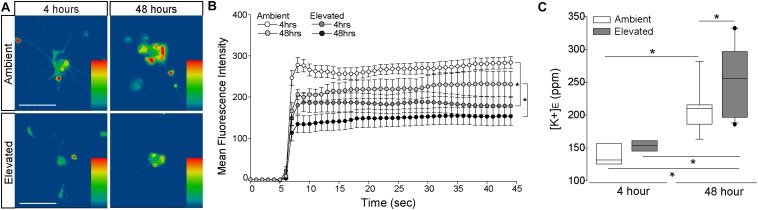
Elevated pressure decreases inward cation flux and increases extracellular K^+^ concentration in RGCs *in vitro*. **(A)** Representative heat maps showing the fluorescent signal of Thallos dye in RGCs exposed to ambient (top) and elevated (bottom) pressure for 4 h (left) or 48 h (right). Images were taken after the addition of thallium. Scale bar = 100 μM. **(B)** Line graph displaying the mean fluorescence intensity of Thallos dye over time for RGCs exposed to ambient or elevated pressure for 4 or 48 h. ^∗^*p* < 0.05 and error bars represent SEM. *n*(AMB4) = 30 cells, *n*(ELEV4) = 27; *n*(AMB48) = 6, and *n*(ELEV48) = 9. **(C)** Extracellular K^+^ concentration in RGC culture media following 4 h (left) or 48 h (right) of ambient (white) or elevated (gray) pressure, as measured by inductively coupled plasma mass spectrometry. ^∗^*p* < 0.05. *n*(AMB48) = 9 wells, *n*(ELEV48) = 12, *n*(AMB4) = 7, and *n*(ELEV4) = 7.

To determine whether pressure-induced decreases in Na/K-ATPase expression are accompanied by changes in cation homeostasis, we quantified inward flux of cations in purified, primary RGCs, using real-time thallium flux imaging (Weaver et al., 2004; Fischer et al., 2018). Thallium acts as a surrogate for cations and a fluorescent signal is generated by thallium binding to a cell-permeable Thallos dye (Weaver et al., 2004). In this assay, increased fluorescent signal indicates opening of cation channels, which are promiscuously permeable to thallium (Weaver et al., 2004). RGC cultures were loaded with Thallos dye following either 4- or 48-h exposure to ambient or elevated pressure. After addition of thallium, fluorescence was imaged in live cells at 1-s intervals for 45 s. Qualitatively, fluorescent intensity of Thallos dye appears reduced in RGCs exposed to 4 or 48 h of elevated pressure, compared to ambient controls (Figure 5A). We quantified the fluorescent intensity/cell at each 1-s time point between ambient and elevated pressure conditions after both 4 and 48 h exposures (Figure 5B). Exposure to 4 h of elevated pressure decreased thallium flux by 22%, as compared to 4 h of ambient pressure (*p* < 0.05; Figure 5B). By 48 h of exposure to elevated pressure, thallium flux decreased further to 33% of that measured in the respective ambient pressure condition (*p* < 0.05; Figure 5B). For RGC cultures maintained at elevated pressure, thallium flux decreased by approximately 19% between 4 and 48 h of exposure (*p* > 0.05; Figure 5B). Time in culture also slightly reduced (6%) thallium flux in RGCs maintained at ambient pressure (*p* > 0.05; Figure 5B). Together, these data indicate that exposure to both 4 and 48 h of elevated pressure reduces inward flux of cations. Furthermore, reductions in cation flux positively correlated with exposure time, such that 48 h of elevated pressure reduced cation flux more than 4 h of elevated pressure.

Reduced inward flux of cations should lead to a reciprocal increase in [K^+^]_*E*_. Thus, we exposed primary, purified RGC cultures to ambient or elevated pressure for either 4 or 48 h and measured [K^+^]_*E*_, using ICP-MS. Exposure to 4 h of elevated pressure did not significantly alter [K^+^]_*E*_, as compared to ambient pressure (Figure 5C). Consistent with our thallium flux imaging, time in culture significantly increased (34%) [K^+^]_*E*_ in RGCs maintained at ambient pressure (*p* < 0.05; Figure 5C). Exposure to 48 h of elevated pressure increased [K^+^]_*E*_ by 18.5%, as compared to ambient pressure at 48 h (*p* < 0.05; Figure 5C) and by 41%, as compared to elevated pressure at 4 h (*p* < 0.05, Figure 5C). Although inward cation flux is diminished after 4 h of elevated pressure, these data suggest that more substantial decreases in cation flux, like that observed after 48 h, are necessary to significantly elevate [K^+^]_*E*_.

### Inhibition of Na/K-ATPase Endocytosis and Degradation Prevents Pressure-Induced Reduction of Inward Cation Flux

**FIGURE 6:**
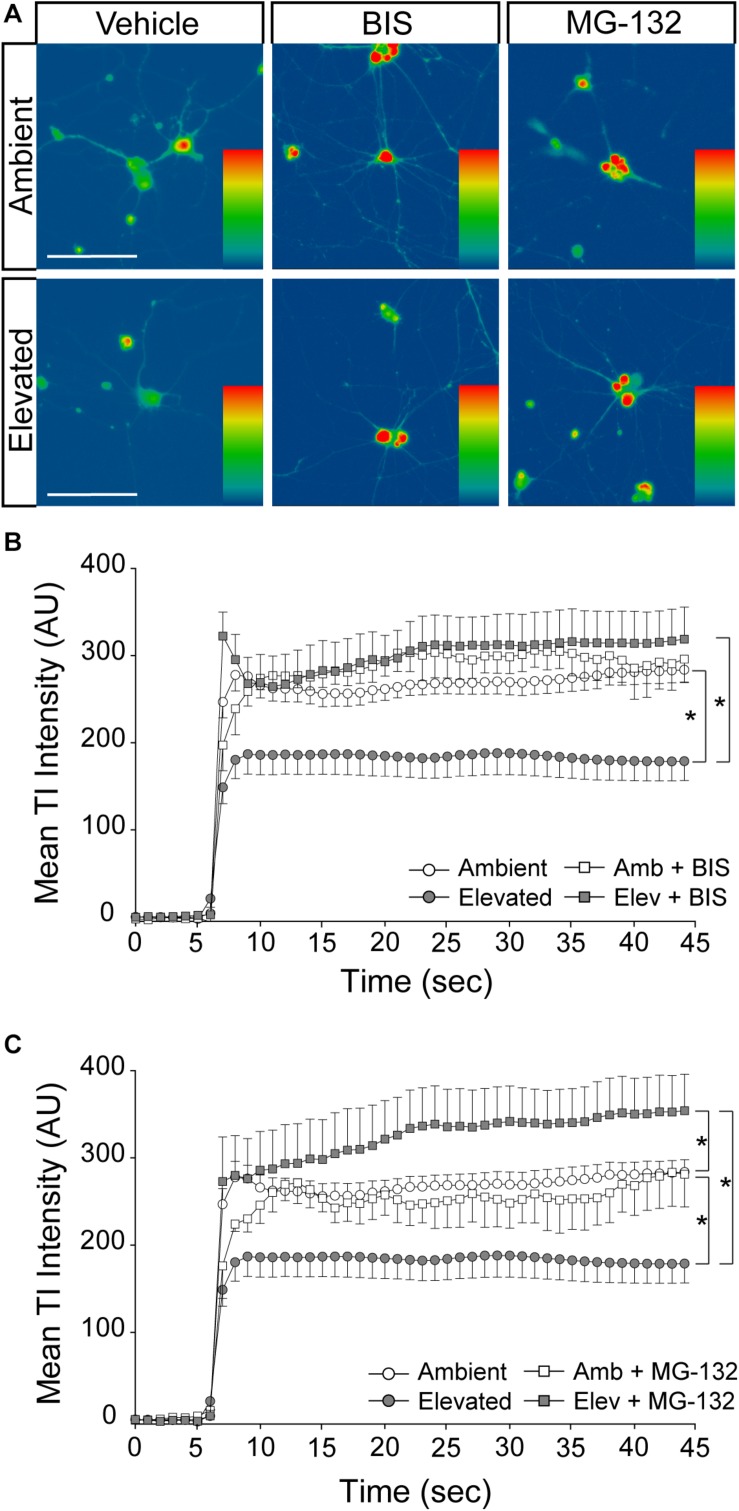
Inhibition of Na/K-ATPase endocytosis and degradation prevents pressure-induced reduction of inward cation flux. **(A)** Representative heat maps showing the fluorescent signal of Thallos dye in RGCs exposed to ambient and elevated pressure for 4 h plus treatment with vehicle, 10 μM bisindolylmaleimide, or 20 μM MG-132. Images were taken after the addition of thallium. Scale bar = 100 μM. **(B)** Line graph displaying the mean fluorescence intensity of Thallos dyes over time for RGCs exposed to ambient or elevated pressure for 4 h plus treatment with vehicle or bisindolylmaleimide. ^∗^*p* < 0.05 and error bars represent SEM. *n*(AMB) = 30 cells, *n*(ELEV) = 27, *n*(AMB + BIS) = 7, and *n*(ELEV + BIS) = 16. **(C)** Line graph displaying the mean fluorescence intensity of Thallos dyes over time for RGCs exposed to ambient or elevated pressure for 4 h plus treatment with vehicle or MG-132. ^∗^*p* < 0.05 and error bars represent SEM. *n*(AMB) = 30 cells, *n*(ELEV) = 27, *n*(AMB + MG-132) = 3, and *n*(ELEV + MG-132) = 13.

To determine whether pressure-induced decreases in Na/K-ATPase expression underlie the reduction in inward cation flux, we treated primary cultures of purified RGCs with vehicle, 10 μM bisindolylmaleimide, or 20 μM MG-132, while exposing them to either ambient or elevated pressure for 4 h. As depicted in Figure 4, inhibition of either Na/K-ATPase endocytosis (bisindolylmaleimide) or degradation (MG-132) is sufficient to retain Na/K-ATPase in the plasma membrane following a 4-h exposure to elevated pressure. Consistent with Figure 5, 4 h of elevated pressure decreased thallium flux by 22% in vehicle-treated cultures, as compared to those maintained at ambient pressure (*p* < 0.05; Figures 6A–C). At ambient pressure, neither bisindolylmaleimide nor MG-132 altered thallium flux, as compared to vehicle (*p* > 0.05; Figures 6A–C). At elevated pressure, bisindolylmaleimide increased thallium influx by 64%, as compared to vehicle treatment at elevated pressure (*p* < 0.05; Figures 6A,B). This increase returned thallium influx to levels comparable to vehicle treatment at ambient pressure (*p* > 0.05; Figures 6A,B). Similarly, treatment with MG-132 increased thallium flux by 78% at elevated pressure, as compared to vehicle-treated cultures exposed to elevated pressure (*p* < 0.05; Figures 6A,C). Interestingly, this increased thallium flux was 39% greater than that observed with vehicle treatment at ambient pressure (*p* < 0.05; Figures 6A,C). These data suggest that inhibition of either Na/K-ATPase endocytosis with bisindolylmaleimide or Na/K-ATPase degradation with MG-132 is sufficient to prevent pressure-induced decreases in inward cation flux.

### Na/K-ATPase Inhibition Reproduces Cation Dyshomeostasis and Repolarization Deficits

If pressure-induced changes in Na/K-ATPase expression underlie cation dyshomeostasis and electrophysiological deficits in RGCs, inhibition of Na/K-ATPase activity alone should mimic these pressure-induced phenotypes. Thus, we examined inward cation flux (thallium flux imaging) and action potential dynamics (patch-clamp physiology) in response to treatment with ouabain, a pharmacological inhibitor of the Na/K-ATPase.

**FIGURE 7:**
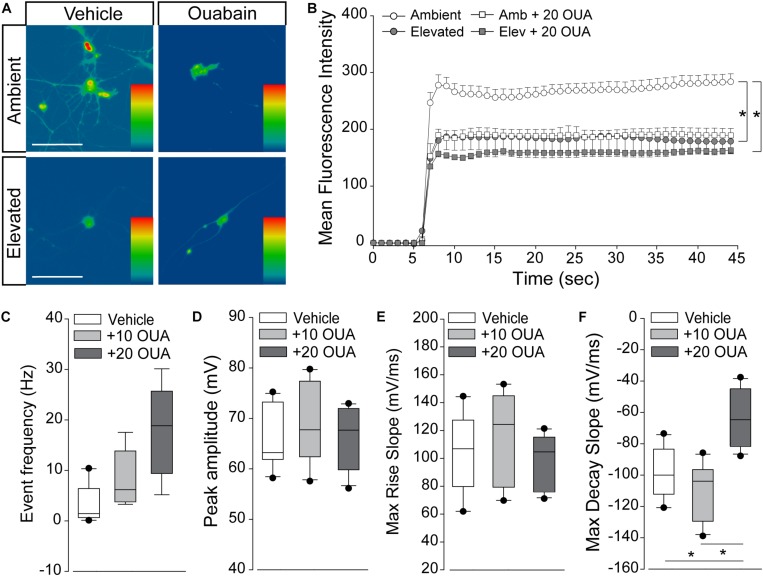
Na/K-ATPase inhibition reproduces cation dyshomeostasis and repolarization deficits. **(A)** Representative heat maps depicting the fluorescent signal of Thallos dye in RGCs exposed to ambient and elevated pressure for 4 h in the presence of vehicle or 20 μm ouabain or vehicle. Scale bar = 100 μM. **(B)** Line graph displaying the mean fluorescence intensity of Thallos dyes over time for RGCs exposed to ambient or elevated pressure for 4 h in the presence of vehicle or 20 μm ouabain. ^∗^*p* < 0.05 and error bars represent SEM. *n*(AMB) = 30, *n*(ELEV) = 27, *n*(AMB + OUA) = 5, and *n*(ELEV + OUA) = 6. **(C–F)** Whole-cell patch-clamp physiology in RGCs from retina of saline-injected eyes following puff administration of vehicle, 10, or 20 μM ouabain. ^∗^*p* < 0.05. **(C)** Frequency of spontaneous spiking depicted as mean event frequency (Hz) across cells for each condition. *n*(vehicle) = 12 cells, *n*(10) = 9, *n*(20) = 9 (from *n* = 3 retina/condition). **(D)** Peak amplitude of spontaneous spiking depicted as mean peak amplitude (mV) across cells for each condition. *n*(vehicle) = 11 cells, *n*(10) = 10, *n*(20) = 11 (from *n* = 3 retina/condition). **(E)** Max rise slope of spontaneous spiking depicted as mean max rise slope (mV/ms) across cells for each condition. *n*(vehicle) = 14 cells, *n*(10) = 10, *n*(20) = 11 (from *n* = 3 retina/condition). **(F)** Max decay slope of spontaneous spiking depicted as mean max decay slope (mV/ms) across cells for each condition. *n*(vehicle) = 10 cells, *n*(10) = 7, *n*(20) = 11 (from *n* = 3 retina/condition).

We exposed primary cultures of purified RGCs to ambient or elevated pressure for 4 h in the presence or absence (vehicle) of 20 μM ouabain and measured cation influx by thallium flux imaging. This concentration of ouabain is known to inhibit Na/K-ATPase activity in neurons from rat brain (Brosemer, 1985; Oselkin et al., 2010). Consistent with Figures 5, 6, exposure to 4 h of elevated pressure decreased thallium flux by 22% in vehicle-treated cultures, as compared to those at ambient pressure (*p* < 0.05; Figures 7A,B). Treatment with ouabain similarly decreased thallium flux by 26% in cultures maintained at ambient pressure, as compared to vehicle-treated cultures (*p* < 0.05; Figures 7A,B). At elevated pressure, ouabain treatment decreased thallium influx by 33%, compared to vehicle-treated cultures at ambient pressure (*p* < 0.05; Figures 7A,B). This decrease was equivalent to that induced by elevated pressure alone (*p* > 0.05; Figures 7A,B). These data indicate that inhibition of the Na/K-ATPase with ouabain is sufficient to reduce inward cation flux to levels similar to that observed with exposure to elevated pressure. Furthermore, additional inhibition of Na/K-ATPase activity in RGCs does not exacerbate pressure-induced reductions in cation influx. This is likely due to the pressure-induced reduction of Na-K/ATPase on the plasma membrane.

Next, we determined whether short-term Na/K-ATPase inhibition could replicate IOP-dependent phenotypes in RGC electrophysiology. Using whole-cell patch-clamp physiology in intact retina, we measured the frequency and amplitude of spontaneous activity in RGCs following puff administration of 10 or 20 μM ouabain. To allow direct comparison with electrophysiological data in Figure 1, we utilized retina 4 weeks after bilateral saline injection. Ouabain induced a slight, non-significant increase in the spike frequency of RGCs at both 10 and 20 μM concentrations (*p* > 0.05; Figure 7C). Ouabain did not alter the peak amplitude of spikes at either concentration, as compared to vehicle treatment (*p* > 0.05; Figure 7D). Similarly, there was no significant difference in the max rise slope (depolarization phase) between vehicle and ouabain treatment (*p* > 0.05; Figure 7E). Administration of 10 μM ouabain did not alter the max decay slope (repolarization phase) in RGCs, as compared to vehicle treatment (*p* > 0.05: Figure 7F). In contrast, 20 μM ouabain increased the max decay slope by 37 and 44%, as compared to vehicle and 10 μM ouabain, respectively (*p* < 0.05 for both; Figure 7F). This increase in the max decay slope is similar in magnitude to that noted after 4 weeks of elevated IOP in the microbead occlusion model (Figure 1). These data suggest that healthy RGCs sufficiently counter brief disruptions in Na/K-ATPase activity to maintain both spike frequency and amplitude. However, more robust perturbation of Na/K-ATPase activity (20 μM ouabain) reproduces delay in the repolarization phase similar to that observed with elevated IOP.

## Discussion

Here, we examined the electrophysiological signature of individual RGCs in glaucomatous retina with respect to their axon transport facility. Our findings indicate that deficits in axon transport reflect measurable deficiencies in the ability of RGCs to maintain spiking frequency. This physiological phenotype appears to arise from elongation of the repolarization phase of the action potential. Elevated pressure reduces Na/K-ATPase activity, first by internalization and degradation and, finally, by transcriptional downregulation. This alteration in Na/K-ATPase activity leads to reduced cation influx in RGCs that ultimately produces cation dyshomeostasis. Pharmacological inhibition of internalization and degradation pathways prevents early decreases in Na/K-ATPase activity, stabilizes cation flux, and prevents K^+^ dyshomeostasis, despite elevated pressure. Furthermore, in healthy RGCs, pharmacological inhibition of the Na/K-ATPase reproduces both cation influx and repolarization phase phenotypes observed with elevated pressure. These findings suggest that (1) deficits in axon transport also likely reflect impaired electrophysiological function of RGCs, (2) failure to maintain electrochemical gradients and resulting cation dyshomeostasis is an early phenotype of glaucomatous pathology in RGCs, and (3) pressure-induced changes in Na-K/ATPase activity strongly contribute to this early electrophysiological impairment.

In retina from eyes with microbead-induced ocular hypertension, whole cell patch-clamp physiology revealed a decrease in both the frequency and amplitude of K^+^-induced spikes that was accompanied by a significant increase in the max decay slope (Figure 1). This increase in the max decay slope indicates flattening of the repolarization phase of the action potential, which results in slower repolarization. Slower repolarization and, thus, impaired preparation for subsequent action potentials can explain decreases in spike amplitude and frequency noted in both current- and KCl-induced recording paradigms. This is consistent with previous studies describing decreased light-evoked spiking and depolarization of Vm in RGCs from eyes with microbead-induced ocular hypertension (Chen et al., 2015; Pang et al., 2015; Risner et al., 2018). To directly assess the impact of ion concentration gradients on the phase dynamics of the RGC action potential, we utilized KCl stimulation instead of light stimulation, which is not selective for the detection of altered electrochemical gradients and the mechanisms that may underlie them.

The repolarization phase of the action potential is defined by the re-establishment of electrochemical gradients. This is accomplished primarily by activity of the Na/K-ATPase, which pumps 3 Na^+^ ions out for every 2 K^+^ ions taken in. We found that 4 weeks of elevated IOP reduces gene and protein expression of both total and α1 Na/K-ATPase in retina from microbead-injected eyes (Figure 2). *In vitro* studies confirmed that elevated pressure reduces protein expression of both total and α1 Na/K-ATPase in RGCs, specifically (Figure 3). Interestingly, this pressure-induced decrease was evident at both 4 and 48 h of exposure, suggesting that the initial decrease in Na/K-ATPase expression is not mediated by genetic downregulation.

Expression of the Na/K-ATPase can be modulated at the protein level by altering translocation of the protein to the plasma membrane or by promoting endocytosis (Chibalin et al., 1999; Alves et al., 2010). There is evidence that cell surface expression of the Na/K-ATPase can be modulated by trafficking of protein between a plasma membrane-associated pool and pool within intracellular compartments that has been internalized by endocytosis (Barlet-Bas et al., 1990; Horisberger and Rossier, 1992; Alves et al., 2010). The Na/K-ATPase undergoes constitutive endocytosis and degradation (Wang et al., 2014). Regulation of Na/K-ATPase cell surface expression can occur via control of this constitutively high rate of internalization and degradation. This process does not require *de novo* protein synthesis and thus is a very efficient mode of regulation allowing rapid adaptation of activity in response to cellular signals (Wang et al., 2014). For example, an increase in the intracellular Na^+^ concentration has been shown to increase Na/K-ATPase cell surface expression by inhibiting p38 kinase-mediated endocytosis, and eventual degradation, of the Na/K-ATPase (Wang et al., 2014). Inhibition of endocytosis with bisindolylmaleimide and inhibition of degradation with MG-132 were both sufficient to retain expression of total Na/K-ATPase after a 4-h exposure to elevated pressure (Figure 4). These data confirmed that elevated pressure induces an early reduction in available Na/K-ATPase via activation of endocytosis and degradation pathways. Phosphorylation of the Na/K-ATPase by PKC or p38 kinase initiates the endocytosis pathway (Chibalin et al., 1998; Lecuona et al., 2009; Wang et al., 2014; Magnani et al., 2017). Following endocytosis, the Na/K-ATPase is degraded by the proteasome (Lecuona et al., 2009; Magnani et al., 2017). One possible mechanism for how elevated pressure induces activation of these pathways is through intracellular Ca^2+^ signaling. PKC is activated by diacylglycerol and intracellular Ca^2+^ (Huang, 1989). One of the earliest events in RGC degeneration during glaucoma is intracellular Ca^2+^ dysregulation (Calkins, 2012; Sappington et al., 2015). Increased intracellular Ca^2+^ levels, via TRPV1 signaling, are found in primary, purified RGC cultures after exposure to only 1 h of elevated pressure (Sappington et al., 2009). Early activation of intracellular Ca^2+^ signaling in response to elevated pressure could activate PKC signaling in RGCs, resulting in activation of the Na/K-ATPase endocytosis pathway. Another possible mechanism is through p38 kinase-mediated endocytosis. Activation of p38 MAPK is evident in multiple mouse models of glaucoma and treatment with a selective inhibitor of the p38 MAPK catalytic domain (Ro3206145) prevents axon transport deficits as well as structural degeneration of RGC axons (Raingeaud et al., 1995; Herlaar and Brown, 1999; Zarubin and Han, 2005).

Influx of K^+^ against its concentration gradient, for example, by the Na/K-ATPase, is an essential aspect of reestablishing electrochemical gradients. Thallium flux imaging revealed that elevated pressure reduces cation flux in RGCs *in vitro* (Figure 5). ICP-MS revealed that pressure-induced decreases in cation influx result in an increase in extracellular K^+^ concentration after 48 h of pressure elevation (Figure 5). It is unlikely that this K^+^ increase is due to increased permeability of the plasma membrane, as we found that 48 h of elevated pressure induces apoptosis, but not cytotoxicity, in RGCs *in vitro* (Supplementary Figure S1). This was confirmed by our pharmacological studies with bisindolylmaleimide and MG-132, demonstrating maintenance of cation influx at ambient levels in RGCs exposed to elevated pressure (Figure 6). Our loss-of-function studies with the Na/K-ATPase inhibitor ouabain suggest that even brief inhibition of Na/K-ATPase in healthy retina can reproduce the repolarization phase phenotype observed in glaucomatous retina. Thus, our data suggest that elevated pressure induces cation dyshomeostasis via reduced inward cation flux secondary to endocytosis and degradation of the Na/K-ATPase. Our transcriptome analysis in the microbead occlusion model further suggests that this cation dyshomeostasis is likely chronic, owing to ultimate downregulation of the Na/K-ATPase at the gene level with prolonged exposure to elevated IOP (Figure 2). Importantly, the electrophysiological deficits we observed were apparent after 4 weeks of microbead-induced IOP elevation. At this time point, RGCs exhibit axon transport deficits with fairly moderate structural degeneration of axons in the optic nerve (Crish et al., 2010; Echevarria et al., 2017; Wareham et al., 2018). Similarly, decreases in cation flux and internalization of the Na-K-ATPase were evident after only 4 h of exposure to elevated pressure. Thus, internalization and degradation of the Na/K-ATPase is likely an early response to elevated pressure that is perpetuated by downregulation of the channel with chronic pressure exposure. Impairment in RGC activity arising from disruptions in the electrochemical gradient could be a significant part of the transition from functional deficits to irreversible, structural loss of RGC axons in the optic nerve. As maintaining proper physiological activity is crucial for neuron survival, these deficits could be a first step toward eventual cell death (Duda et al., 2016).

Based on our *in vitro* findings, preventing internalization and degradation of the Na/K-ATPase is a promising direction for a therapeutic to correct pressure-induced decreases in cation influx. Future directions for these studies will include finding an approach to therapeutically target Na/K-ATPase internalization and downregulation in an *in vivo* system. While bisindolylmaleimide and MG-132 were suitable options for *in vitro* studies, they may not be the optimal choice for *in vivo* studies due to off-target effects on other cell types in the eye. Additionally, long-term overexpression of the Na/K-ATPase could have negative consequences. Global overexpression is not a feasible option as the Na/K-ATPase is expressed throughout the cardiovascular and renal systems and retina-specific overexpression could result in compensatory mechanisms to adjust for altered representation of the Na/K-ATPase. Therefore, finding a suitable *in vivo* pharmacological approach to target the Na/K-ATPase is an important next step to elucidating the role of the Na/K-ATPase in RGC electrophysiological dysfunction during glaucoma.

In conclusion, our findings indicate that failure of electrochemical gradients and resulting cation dyshomeostasis is as an early phenotype of glaucomatous pathology in RGCs that may have significant bearing on efforts to restore RGC health in diseased retina. Our data clearly demonstrate a link between compromised axon transport, impaired physiological activity, and Na/K-ATPase-mediated deficiencies in electrochemical homeostasis. However, it is unclear whether pressure-induced reductions in Na/K-ATPase activity are an etiological factor or a response to RGC compromise. As outlined above, elevated pressure could initiate intracellular Ca^2+^ signaling, resulting in PKC activation, phosphorylation of the Na/K-ATPase, and Na/K-ATPase internalization and downregulation concurrent with mechanisms that produce impairment in axon transport (Huang, 1989; Sappington et al., 2009, 2015; Calkins, 2012). Alternatively, Na/K-ATPase internalization and downregulation could be a response to RGC compromise and serve as an element of targeted degeneration. In either case, impairment of electrochemical gradients has the potential to impact not only the RGC exhibiting the deficit but also surrounding RGCs, which must also maintain electrochemical gradients to properly function. Our findings raise the interesting possibility for a “snowball” effect, where prolonged disruption of electrochemical gradients in a small cluster of RGCs could lead to cation dyshomeostasis that impairs the electrochemical gradients of RGCs surrounding that cluster and so on. Thus, the electrophysiological phenotypes and mechanisms described here may be relevant for topographic spread of neurodegeneration in glaucoma.

## Data Availability Statement

The RNA sequences generated in this study can be found in the NCBI’s Gene Expression Omnibus and are accessible through GEO series accession number GSE116915 (https://www.ncbi.nlm.nih.gov/geo/query/acc.cgi?acc=GSE116915).

## Ethics Statement

The animal study was reviewed and approved by the Institutional Animal Care and Use Committee of Vanderbilt University Medical Center.

## Author Contributions

RF, MR, AR, and LW performed the experiments and analyzed the data. RF and RS designed the experiments, interpreted the results, and wrote the manuscript. All authors have read and approved the final manuscript.

## Conflict of Interest

The authors declare that the research was conducted in the absence of any commercial or financial relationships that could be construed as a potential conflict of interest.

## Footnotes

1 https://www.ncbi.nlm.nih.gov/geo/query/acc.cgi?acc=GSE116915
