# Supplementary figures and images for: Impairment of Membrane Repolarization Accompanies Axon Transport Deficits in Glaucoma
Source: Front Neurosci. 2019 Nov 1;13:1139. doi: 10.3389/fnins.2019.01139 (PMC6838637; doi:10.3389/fnins.2019.01139)

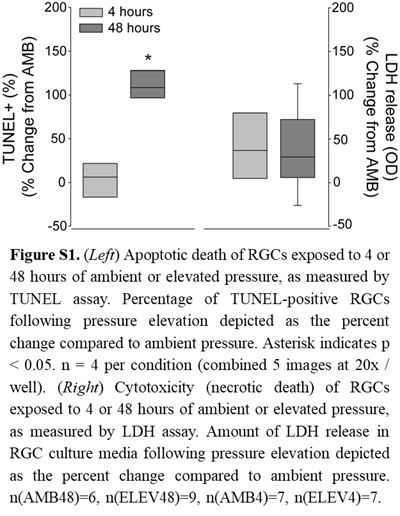

Supplement: Supplementary file 1 [file Image_1.tif]
